# Superficial low‐grade fibromyxoid sarcoma

**DOI:** 10.1111/cup.14325

**Published:** 2022-11-02

**Authors:** Shira Ronen, Jennifer S. Ko, Brian P. Rubin, Scott E. Kilpatrick, Wei‐Lien Wang, Alexander J. Lazar, John R. Goldblum, Steven D. Billings

**Affiliations:** ^1^ Department of Pathology Cleveland Clinic Cleveland Ohio USA; ^2^ Department of Pathology The University of Texas MD Anderson Cancer Center Houston Texas USA

**Keywords:** Evans tumor, immunohistochemistry, low‐grade fibromyxoid sarcoma, skin neoplasms, superficial

## Abstract

**Background:**

Low‐grade fibromyxoid sarcoma (LGFMS) typically involves deep soft tissue (beneath the fascia) of the proximal extremities and trunk. Long‐term follow‐up has shown a high rate of local recurrence, metastasis, and death. To the best of our knowledge, there is only one previous large series focusing on superficial LGFMS suggesting superficial tumors are disproportionately more common in children and may have a better prognosis. Our study's primary goals are to confirm these findings and increase general awareness that LGFMS may arise in superficial soft tissue.

**Methods:**

We retrieved our cases of superficial LGFMS diagnosed between 2008 and 2020. Available slides were reviewed, and clinical data and follow‐up information were obtained.

**Results:**

The patients included nine males and 14 females with a median age of 29 years; eight (35%) were children (<18 years) and five (22%) were young adults (18–30 years). The majority involved the lower extremities (65%). The tumors were primarily centered in the subcutis (91%) and dermis (9%). Microscopically, they had typical features of LGFMS with alternating fibrous and myxoid zones composed of bland, slightly hyperchromatic spindled cells. All were positive for MUC4 by immunohistochemistry and/or *FUS* rearrangement by FISH. Follow‐up on 14 cases ranged from 11 to 148 months (median 61 months) with no evidence of recurrences or distant metastases.

**Conclusions:**

Compared to conventional deep‐seated counterparts, superficial LGFMS is more likely to occur in the extremities of children and young adults and may have a better clinical outcome. Further studies with longer follow‐up will likely help support these findings.

## 1 INTRODUCTION

Low‐grade fibromyxoid sarcoma (LGFMS) is a distinctive malignant fibroblastic neoplasm characterized by alternating fibrous and myxoid areas containing deceptively bland spindled cells classically exhibiting a short fascicular and whorled growth pattern.^1^, ^2^, ^3^ The spectrum includes cases with giant rosettes, originally designated as “hyalinizing spindle cell tumor with giant rosettes.”^4^, ^5^ These tumors consistently have either *FUS::CREB3L2* or *FUS::CREB3L1* gene fusions, and rarely *EWSR1::CREB3L1*.^6^ Multiple studies have shown recurrence rates of 1%–9% and metastasis in 6%–27%, primarily to lungs, pleura, and chest wall.^4^, ^7^, ^8^, ^9^ However, these rates are higher with long‐term follow‐up; Evans's study with the longest follow‐up (at least 5 years) reported local recurrence of 64% (up to 15 years after diagnosis), metastases in 45% (up to 45 years after diagnosis), and death of disease in 42% (from 3 to 42 years after diagnosis).^2^

LGFMS typically presents as a slowly growing asymptomatic mass on the lower extremities, usually the thigh, followed by the groin/perineum and trunk. Most lesions are localized to the deep soft tissues, including the skeletal muscle.^1^, ^5^ Only one previous large series focusing on superficial LGFMS suggested superficial tumors were disproportionately more common in children and might have a better prognosis, but this study was limited by the lack of available confirmatory testing at the time (e.g., MUC4 immunohistochemistry).^3^ Herein, we report an additional 23 cases of superficial LGFMS in order to confirm these findings and increase general awareness of superficial LGFMS.

## 2 MATERIALS AND METHODS

The Institutional Review Board Committee of the authors' institutions approved this study. Our electronic surgical pathology files were retrospectively reviewed to identify all cases of LGFMS diagnosed from January 2008 to January 2021. For inclusion, the tumors had to be confined to superficial soft tissue (dermis and/or subcutis only) without fascial or skeletal muscle involvement. Twenty‐three cases met the criteria, of which 16 were consultation cases. Demographic and clinical information, clinical diagnosis, imaging studies, and follow‐up were obtained from medical records or by communication with referring pathologists and primary physicians. The cutoff to be considered a pediatric patient was ≤18 years. Histopathologic parameters including location, mitotic figures, borders of the tumor, and presence of necrosis were collected. Results of immunohistochemical stains and molecular studies for *FUS* rearrangement by FISH were obtained.

## 3 RESULTS

### 3.1 Clinical data

The clinicopathologic features are summarized in Table 1. Twenty‐three patients (nine males; 14 females) with a median age of 29 years (range: 2–65 years) constituted the cohort. Eight (35%) patients were children and five (22%) were young adults (18–30 years). The majority involved the lower extremity (65%), including the gluteal region (five cases), thigh (four cases), inguinal region (three cases), leg, great toe, and pretibial location (one each). The remaining sites included flank (three cases), occipital scalp (two cases), abdominal wall, axilla, and paraspinal (one each). The lesions range from 1 to 9.2 cm in greatest dimension (median of 2.8 cm). When available, the pre‐operative clinical impression was mainly benign, with cyst being the most common diagnosis followed by lipoma, nodular fasciitis, pilomatricoma, hematoma, peripheral nerve sheath tumor, and vascular malformation. In three patients (Cases 1, 7, and 12), the clinical differential diagnosis included malignant entities: metastatic squamous cell carcinoma, synovial sarcoma, and sarcoma not otherwise specified. A contributor's histopathologic diagnosis was provided for four cases and included LGFMS, solitary fibrous tumor (SFT), calcifying fibrous pseudotumor, fibrohistiocytic lesion, and spindle cell/myxoid lipoma.

**TABLE 1:** Clinicopathologic data on superficial LGFMS

Case no.	Age (y)/sex	Site	Size (cm)	Location of tumor	Necrosis	Mitotic figures	Margin status on excision specimen	Circumscribed/infiltrative	Confirmatory test^a^	Follow‐up (mo)	Outcome
1	52/M	Inguinal region	2.7	Subcutis	NI	NI	P	Circumscribed	*FUS* FISH	99	NED
2	7/F	Thigh at inguinal crease	2.4	Subcutis	NI	1/10 HPF	N	Circumscribed	*FUS* FISH	148	NED
3	45/M	Abdominal wall	9.2	Subcutis	NI	NI	P	Circumscribed	*FUS* FISH	93	NED
4	4/F	Flank	2	Subcutis	NI	1/10 HPF	P	Circumscribed	*FUS* FISH	132	NED
5	23/F	Groin	Unknown	Subcutis	NI	NI	P	Circumscribed	*FUS* FISH	LTF	LTF
6	46/M	Perianal	2.8	Dermis	NI	NI	N	Circumscribed	MUC4 IHC and *FUS* FISH	LTF	LTF
7	41/F	Leg	1.8	Subcutis	NI	1/10 HPF	N	Circumscribed	MUC4 IHC	80	NED
8	57/F	Buttock	3	Subcutis	NI	NI	P	Circumscribed	MUC4 IHC	LTF	LTF
9	19/F	Axilla	1	Subcutis	NI	6/10 HPF	P	Circumscribed	MUC4 IHC	LTF	LTF
10	56/M	Buttock	4	Subcutis	NI	NI	Not available	Cannot be assessed	MUC4 IHC	LTF	LTF
11	61/F	Gluteal region	4.5	Subcutis	NI	NI	N	Circumscribed	MUC4 IHC	61	NED
12	12/F	Pretibial	3.3	Subcutis	NI	NI	N	Circumscribed	MUC4 IHC	61	NED
13	4/F	Gluteal region	2	Subcutis	NI	NI	N	Circumscribed	MUC4 IHC	11	NED
14	7/M	Thigh	2.8	Subcutis	NI	NI	N	Cannot be assessed	MUC4 IHC and *FUS* FISH	48	NED
15	29/M	Flank	4.2	Subcutis	NI	NI	N	Circumscribed	MUC4 IHC	30	NED
16	15/F	Thigh	1.6	Subcutis	NI	NI	P	Circumscribed	MUC4 IHC	LTF	LTF
17	29/M	Paraspinal	5	Subcutis	NI	NI	N	Infiltrative	MUC4 IHC	30	NED
18	50/M	Flank	5	Subcutis	Focal	NI	P	Cannot be assessed	MUC4 IHC	LTF	LTF
19	2/M	Great toe	1	Dermis	NI	NI	N	Circumscribed	MUC4 IHC	13	NED
20	23/F	Occipital scalp	2	Subcutis	NI	NI	N	Circumscribed	MUC4 IHC	LTF	LTF
21	8/F	Occipital scalp	Unknown	Subcutis	NI	1/10 HPF	P	Cannot be assessed	MUC4 IHC	LTF	LTF
22	65/F	Thigh	5	Subcutis	NI	NI	P	Cannot be assessed	*FUS* FISH	15.7	Death unrelated to LGFMS
23	49/F	Groin	3	Subcutis	NI	NI	N	Circumscribed	*FUS* FISH	61.9	NED

Abbreviations: F, female; HPF, high‐power field; LGFMS, Low‐grade fibromyxoid sarcoma; LTF, lost to follow‐up; M, male; mo, months; N, negative; NED, no evidence of disease; NI, not identified; P, positive; y, years.

^a^ Confirmatory test by MUC4 immunohistochemistry or FISH for *FUS* rearrangement.

Follow‐up available on 14 cases ranged from 11 to 148 months (median 61 months). None developed recurrence or documented metastases. One patient died from metastatic ovarian cancer.

### 3.2 Pathologic features

**FIGURE 1:**
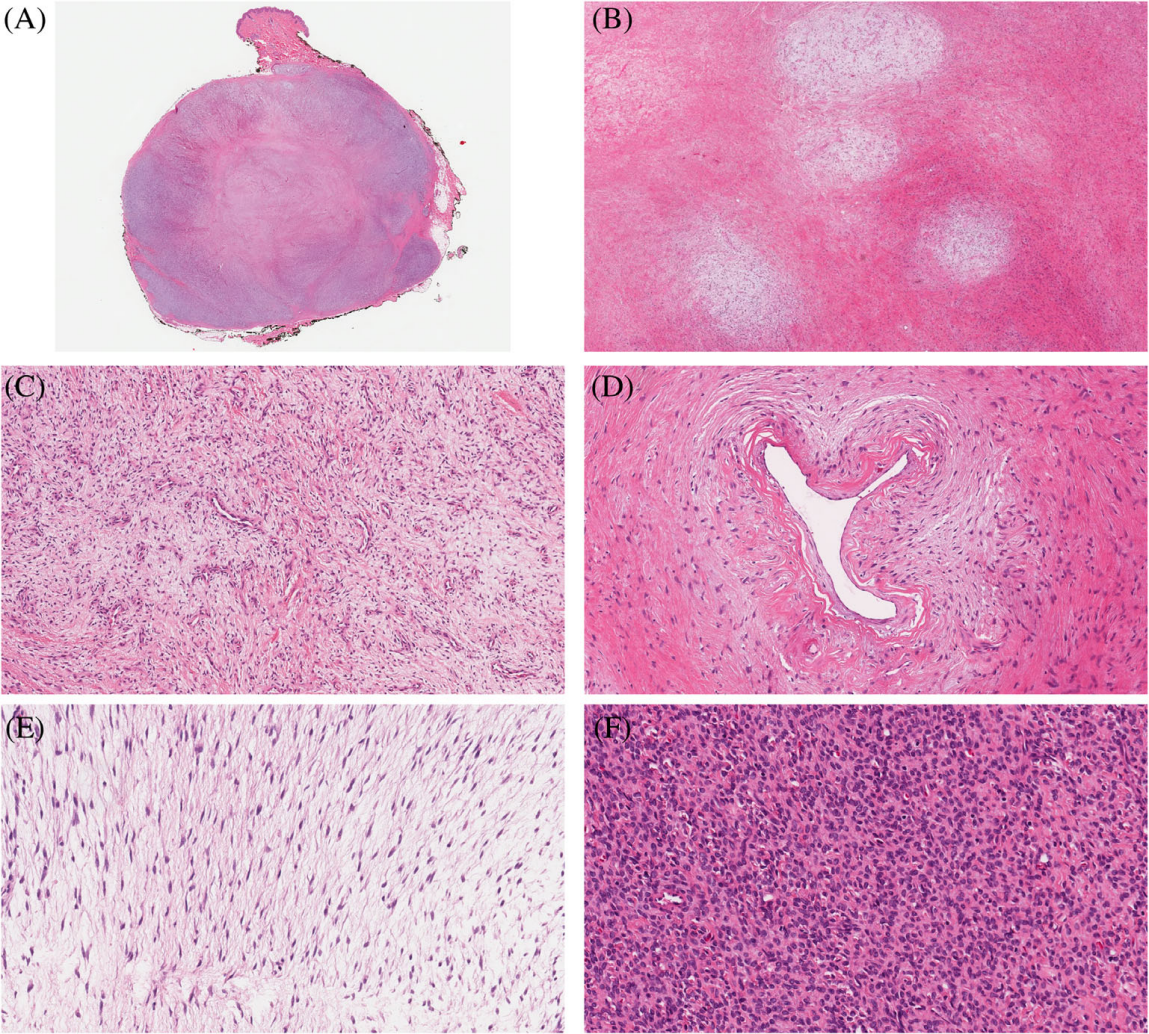
Superficial low‐grade fibromyxid sarcoma. Superficial low‐grade fibromyxoid sarcoma is confined to the superficial soft tissue without fascial or skeletal muscle involvement—Case 4 (A). There are alternating fibrous and myxoid zones with abrupt transition with a swirling, whorled growth pattern—Case 15 (B). The tumor vasculature consists of small branching to curvilinear vessels—Case 1 (C). There were also arteriole‐sized vessels with perivascular sclerosis—Case 15 (D). The neoplastic cells are very bland with spindled morphology in fibromyxoid stroma—Case 2 (E). There were hypercellular areas with sheets of cells with moderate pleomorphism—Case 12 (F). (H&E; A, scanned image; B, ×25; C and D, ×100; E and F, ×200)

**FIGURE 2:**
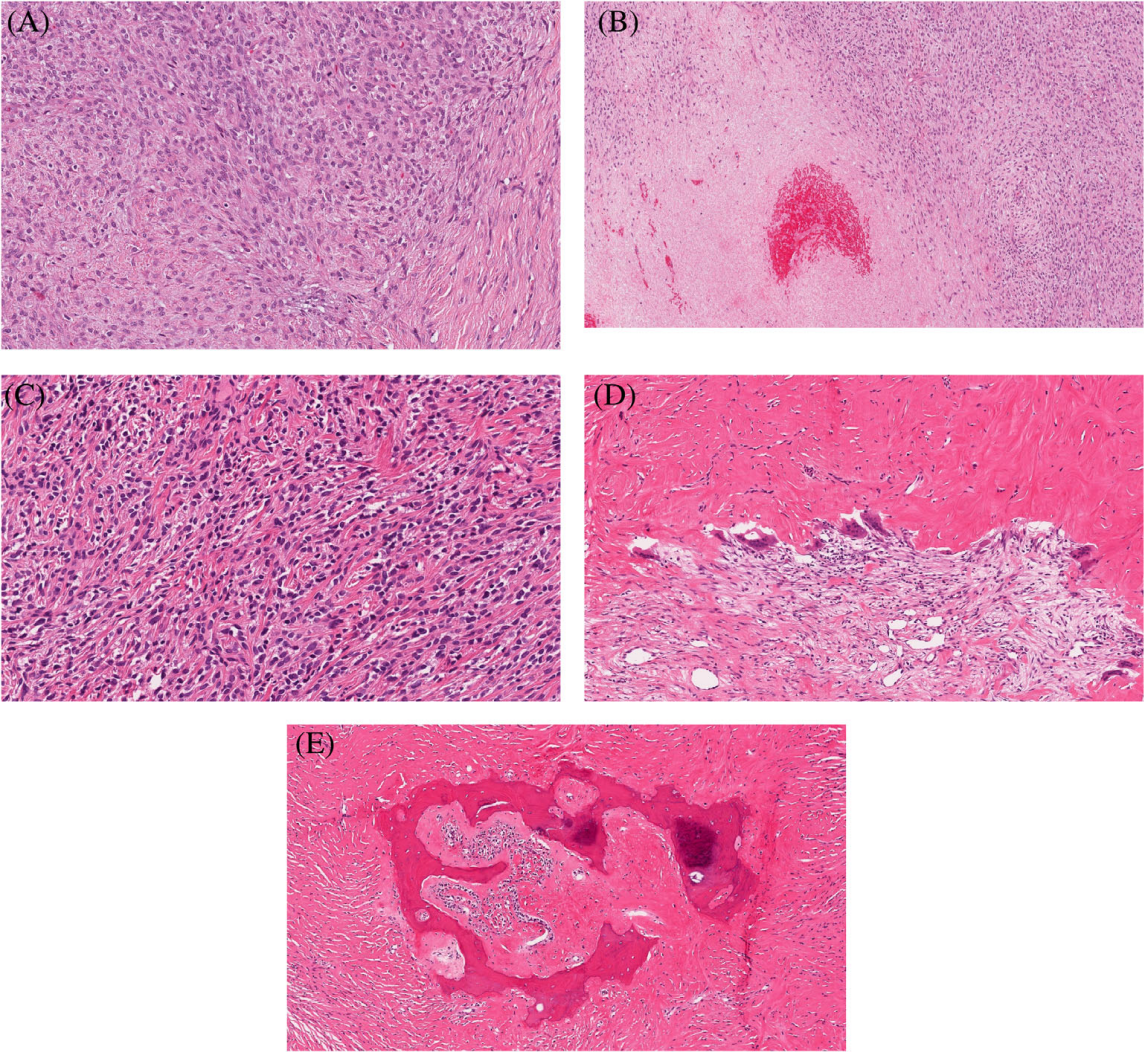
Unusual features of superficial low‐grade fibromyxoid sarcoma. Case 9 shows an increased number of mitotic figures and increased cellularity with mild pleomorphism and vaguely stroriform pattern (A). There is also an area of coagulative necrosis (B). Case 11 displays a prominent hyalinized sclerotic collagen matrix that is associated with bland cells in vague cords resembling sclerosing epithelioid fibrosarcoma (C). This case also exhibits multiple multinucleated giant cells (D) with osseous metaplasia (E). (H&E; A, ×200; B, ×100; C, ×200; D, ×100; E, ×80)

Histopathologically, the tumors were primarily centered in the subcutis (21/23; 91%), with two centered in the dermis (2/23; 9%) (Figure 1A). The majority were circumscribed (17/18; 94%) with eight having a thick fibrous pseudocapsule (8/18; 44%). All had classic features of LGFMS characterized by alternating areas of a collagenized stroma admixed with myxoid zones, both containing a population of bland spindled cells displaying a storiform to whorled growth pattern (Figure 1B). A prominent vascular network of curvilinear to arborizing vessels was more prominently observed in the myxoid zones (Figure 1C). Perivascular sclerosis or hypercellularity was noted in some of the cases (Figure 1D). The tumor cells had bland, oval to spindled, slightly hyperchromatic nuclei (Figure 1E). The cellularity of the lesions varied widely from low to moderate, with rare cases showing hypercellular areas with sheets of cells and mild to moderate nuclear pleomorphism (Figure 1F). Significant pleomorphism was absent, and necrosis was seen in only one tumor (1/23; 4%). Mitotic figures were mostly lacking (18/23; 78%) and, when present (4/23; 17%), scarce with one per 10 high‐power fields. Only one case (case nine) had conspicuous mitotic activity (six mitotic figures per 10 high‐power fields) (Figure 2A). This same case also exhibited non‐perivascular hypercellular areas with more round cells, mild‐to‐moderate pleomorphism, a vaguely storiform growth pattern, and focal necrosis (Figure 2C). Case 11 showed morphologic patterns of both LGFMS and sclerosing epithelioid fibrosarcoma (SEF) with prominent hyalinized sclerotic collagen matrix associated with bland epithelioid cells arranged in vague cords (Figure 2D). This case also displayed unusual features, including multinucleated giant cells and osseous metaplasia (Figure 2E,F). Collagen rosettes were not identified in any case. Of 23 cases, 10 showed a positive margin at the excision specimen. Case 10 was a biopsy and information on excision margin status is not available.

**FIGURE 3:**
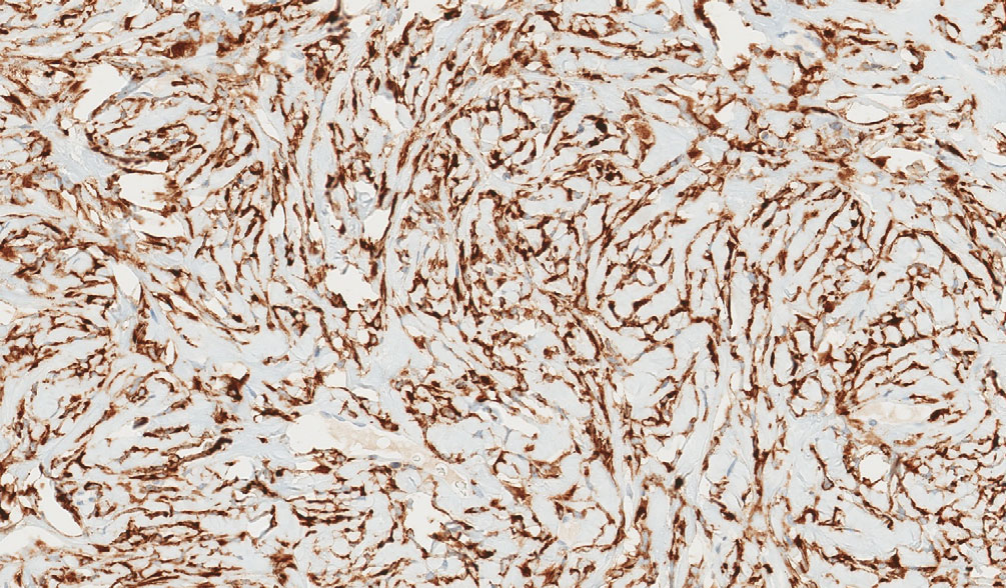
An immunohistochemical for MUC4 shows diffuse and strong positivity within the lesional cells (IHC; ×200).

Immunohistochemical stains for MUC4 showed strong and diffuse positivity in 16/16 cases tested (Figure 3). The remaining seven cases, including two that were also strongly and diffusely positive for MUC4 by immunohistochemistry, showed *FUS* rearrangement by fluorescent in situ hybridization (FISH). Additional immunohistochemical stains performed showed positive expression for Bcl‐2 (2/2) and variable expression for smooth muscle actin (SMA, 2/14) and epithelial membrane antigen (EMA, 1/9). The neoplastic cells were uniformly negative for S100 protein (18/18), CD34 (10/10), desmin (8/8), CD99 (4/4), STAT6 (4/4), beta‐catenin (4/4), pankeratin (3/3), neurofilament (2/2), CD68 (2/2), cytokeratin 903 (1/1), CD56 (1/1), HMB45 (1/1), CD117 (1/1), SOX10 (1/1), Melan‐A (1/1), glial fibrillary acidic protein (GFAP, 1/1), Factor XIIIA (1/1), and CD163 (1/1).

## 4 DISCUSSION

LGFMS is a rare, histopathologically low‐grade sarcoma first described by Dr Harry L. Evans in 1987.^10^ LGFMS accounts for fewer than 5% of soft tissue sarcomas.^7^ It equally involves men and women and typically affects young adults (mean age 35–45 years), but can be seen in patients of any age.^5^, ^11^ The tumor usually arises in the proximal extremities or trunk, but rare locations, including the abdominal and thoracic cavity, visceral organs, and intracranial sites, have been reported.^7^, ^12^, ^13^ The majority occur in a subfascial location, but superficial cases have been described.^3^, ^14^ Our current study describes 23 additional patients with superficial LGFMS confirmed by immunohistochemistry or FISH, promoting awareness for this tumor and expanding upon its clinicopathologic features.

LGFMS classically has alternating hypercellular and hypocellular areas of tumor cells in the background of collagenous, myxocollagenous, or myxoid stroma.^1^ There is usually an abrupt transition between collagenous and myxoid zones. The lesional cells are bland, with small angulated nuclei, scant wispy cytoplasm, and limited nuclear atypia, and are arranged in swirling growth patterns.^1^, ^15^ Mitotic figures are scarce to absent. Curvilinear blood vessels characterized by long, sinuous vessels with a collapsed lumen, with perivascular sclerosis are observed, particularly in the myxoid areas. The tumors typically had classic histopathologic features of LGFMS. In addition, a few cases showed other morphologic variations described in the literature, including a loose storiform pattern, hypercellular areas with more round cells and increased mitotic activity, multinucleated giant cells, and osseous metaplasia.^2^, ^3^, ^15^ One case (Case 11) also had overlapping features of SEF in addition to classic LGFMS features.

SEF was first described by Dr Meis‐Kindblom in 1995 and is characterized by cords and strands of large epithelioid cells with clear or eosinophilic cytoplasm surrounded by densely sclerotic stroma.^16^ In some cases, it can show areas indistinguishable from LGFMS and rarely harbor *FUS* rearrangements suggesting a biological relationship between the two entities.^9^ Studies have also illustrated SEF‐like areas in some cases of LGFMS, as seen in one of our cases.^2^, ^5^, ^9^, ^17^ Despite the overlapping morphologic, immunophenotype, and molecular features, SEF is still considered a separate entity in the current WHO classification. In contrast to LGFMS, SEF has more diverse and complex molecular findings, occurs in somewhat older patients (median age of 48 years), and is more aggressive with a higher death rate and shorter survival.^2^, ^16^, ^18^, ^19^, ^20^ A study raised the possibility that tumors with focal sclerosing‐epithelioid‐sarcoma‐like areas should be regarded as within the spectrum of LGFMS because their study showed that pure SEF (tumors that lack recognizable LGFMS‐like areas) do not usually harbor *FUS* rearrangement.^18^ This distinction provided a strong genetic basis.^21^ Our case with sclerosing‐epithlioid‐sarcoma‐like areas did occur in an older patient that had no evidence of disease with limited follow‐up of 61 months.

The cytogenetic hallmark of LGFMS is t(7;16)(q33;p11), resulting in oncogenic fusion gene of *FUS::CREB3L2* (cAMP‐responsive element‐binding protein 3‐like 2) seen in 75%–95% of cases and t(11;16)(p11;p11) resulting in *FUS::CREB3L1* fusion genes seen in approximately 5% of patients.^6^, ^11^, ^22^, ^23^ Furthermore, two cases with similar morphologic features to the classic LGFMS were found to harbor a novel *EWSR1::CREB3L1* gene fusion.^24^ This translocation was previously shown in two LGFMS‐SEF hybrid cases.^25^ LGFMS exhibit strong and diffuse granular cytoplasmic immunoreactivity with immunohistochemical stains for MUC4. The *MUC4* gene is one of the top upregulated genes in LGFMS located on the long arm of chromosome three (3q29).^26^ MUC4 protein is a high‐molecular‐weight transmembrane glycoprotein that functions in cell growth signaling pathways through interactions with the ERBB2 (HER2) family.^27^ It is normally expressed on many epithelial surfaces, including respiratory and colonic epithelium, where it is believed to have a protective role.^28^ MUC4 is a highly sensitive marker for the diagnosis of LGFMS.^26^ In our study, 16/16 were positive for MUC4. It is important to note that aberrant expression or overexpression of MUC4 has also been reported in various carcinomas, including pancreas, ovary, lung, breast, colon, prostate, and myoepithelial carcinoma, and in mesenchymal tumors, including SEF, synovial sarcoma, ossifying fibromyxoid tumor, epithelioid gastrointestinal stromal tumors, and *PAX3/7::FOXO1* fusion‐positive rhabdomyosarcomas.^25^, ^27^, ^29^, ^30^, ^31^, ^32^ With the exception of MUC4, other immunohistochemical stains are non‐specific in diagnosing LGFMS. EMA positivity has been the most consistent finding with expression ranging from 43% to 91%.^9^, ^11^ Our study shows only 11% (1/9) of the cases positive for EMA. CD99 and Bcl‐2 expression has also been shown in the majority of LGFMS.^9^ In our series, 2/2 cases were positive for BCL2 and 4/4 cases were negative for CD99. The tumor can show variable expression of cytokeratins, CD34, desmin, SMA, claudin 1, and muscle‐specific actin and is consistently negative for S100 protein, Kit, and GFAP.^15^

The largest series to date focusing on superficial LGFMS consists of 19 cases,^3^ but this series predated routinely available confirmatory testing. Besides that series, there were only a few previous studies that included cases of superficial LGFMS, but those articles had too few patients or did not separate superficial from deep LGFMS.^1^, ^33^ The patients in that study included 12 males and seven females with a mean age of 29 years; 37% (7/19) of patients were children^3^ with the lower extremity being the most common location. Their study reported 14/16 (88%) patients with no evidence of disease recurrence and 2/16 (12%) patients with local recurrence at 5 and 16 months (mean follow‐up of 44 months) but no distant metastasis.^3^ Our series, consisting of LGFMS confirmed by ancillary tests, largely corroborates these previous findings: 35% of cases occurred in children, most commonly involving the lower extremity, and none of the patients developed metastasis. In contrast, this series had no episodes of local recurrence and had a modest female predominance (61%). The difference of sex predilection may reflect bias in the original series of superficial LGFMS, as the majority of cases in that series were from the Armed Forces Institute of Pathology. Our data further suggest that superficial LGFMS may have a better overall prognosis than deep LGFMS, likely the result of early recognition of smaller lesions that are amenable to complete excision. It is also well known that children, in contrast to adults, are inclined to have an overall better outcome with low‐grade sarcomas. However, longer‐term follow‐up is still necessary to confirm this, given the propensity for late metastasis in deep LGFMS.

Given the rarity of the tumor, bland cytology, and variable morphology, LGFMS can be difficult to distinguish from some benign mesenchymal tumors and other low‐grade sarcomas. An accurate diagnosis of LGFMS is essential because these patients require complete excision and long‐term follow‐up.

Perhaps the closest histopathologic simulant is perineurioma.^34^, ^35^ Like LGFMS, perineuriomas have bland spindled morphology, whorled pattern, often have variably collagenous to myxoid stroma, and may exhibit collagen rosettes.^3^ Unlike LGFMS, they typically lack a prominent vasculature and abrupt transitions from collagenous to myxoid areas. Immunohistochemical stains can help differentiate the two diagnoses. While both LGFMS and perineurioma may exhibit immunoreactivity for EMA and claudin‐1, perineuriomas are negative for MUC4.^15^, ^26^

One of the benign entities considered by the contributing pathologist was spindle cell lipoma, especially the “low‐fat” and “fat‐free” variants.^36^ Although both tumors present as superficial lesions, spindle cell lipoma is usually present on the upper truck/neck of older men and is extremely rare in the lower extremity.^36^ None of our cases were present on the upper truck/neck. Additionally, in spindle cell lipoma, the spindle cells are characterized by parallel arrays of ropy collagen, and most cases have a significant amount of admixed mature adipocytes. Both lesions can have CD34^+^ cells, but spindle cell lipoma usually has strong CD34 expression.

Superficial fibromatosis most commonly involves the hands or feet. Only one of our cases involved an acral site. Superficial fibromatosis typically lacks myxoid stroma, has a more fascicular growth pattern, and is negative for MUC4.

Cutaneous myxomas, also known as superficial angiomyxoma, are characterized by the presence of bland spindle cells in an abundant myxoid stroma without alternating collagenous zones.^37^, ^38^ Myxomas may also have stromal neutrophils and follicular induction, features not seen in superficial LGFMS. Cutaneous myxomas are negative for MUC4.

Nodular fasciitis may have myxoid stroma, but is overall more cellular than superficial LGFMS with stromal edema, granulation‐tissue‐like vascular proliferation, extravasated red blood cells, and inflammatory cells.^3^ It also has SMA immunoreactivity.

SFT is characterized by a spindle cell proliferation that is often very bland‐appearing and devoid of cytologic atypia against a background of variable degrees of collagenized stroma. Prominent dilated and branching blood vessels in the fibrous component of LGFMS can resemble SFT. However, SFT is negative for MUC4, harbors the *NAB2::STAT6* gene fusion, and is positive for STAT6 by immunohistochemistry.^26^, ^39^

Myxoid DFSP frequently has areas of conventional DFSP in up to 60% of cases.^40^ Myxoid DFSP still has the honeycomb pattern of fat infiltration, has more delicate vessels, and is positive for CD34 and negative for MUC4.^15^, ^41^

In summary, we present a large series of superficial LGFMS and confirm the findings that children are disproportionately affected by superficial LGFMS, and that superficial LGFMS may be less aggressive than deep LGFMS. It can be challenging to separate from other benign entities and, therefore, should be in the histopathologic differential diagnosis of bland spindled cell tumor of the dermis and subcutaneous tissue. The main limitation of our study is the somewhat short follow‐up (range: 11 months to 12.3 years and median of 61 months). Further studies with longer follow‐up would help support these findings.

## CONFLICT OF INTEREST

The authors declare no conflict of interest.

## Data Availability

The data that support the findings of this study are available from the corresponding author upon reasonable request.
